# The use of social networking platforms for sexual health promotion: identifying key strategies for successful user engagement

**DOI:** 10.1186/s12889-015-1396-z

**Published:** 2015-02-06

**Authors:** Hilary J Veale, Rachel Sacks-Davis, Emma RN Weaver, Alisa E Pedrana, Mark A Stoové, Margaret E Hellard

**Affiliations:** Centre for Population Health, Burnet Institute, 85 Commercial Road, Melbourne, VIC 3004 Australia; Department of Epidemiology and Preventive Medicine, Monash University, Melbourne, VIC Australia; The Alfred Hospital, Melbourne, VIC Australia

**Keywords:** Health promotion, Internet, Social networking sites, Sexual health, Facebook, Twitter

## Abstract

**Background:**

Online social networking platforms such as Facebook and Twitter have grown rapidly in popularity, with opportunities for interaction enhancing their health promotion potential. Such platforms are being used for sexual health promotion but with varying success in reaching and engaging users. We aimed to identify Facebook and Twitter profiles that were able to engage large numbers of users, and to identify strategies used to successfully attract and engage users in sexual health promotion on these platforms.

**Methods:**

We identified active Facebook (n = 60) and Twitter (n = 40) profiles undertaking sexual health promotion through a previous systematic review, and assessed profile activity over a one-month period. Quantitative measures of numbers of friends and followers (reach) and social media interactions were assessed, and composite scores used to give profiles an ‘engagement success’ ranking. Associations between host activity, reach and interaction metrics were explored. Content of the top ten ranked Facebook and Twitter profiles was analysed using a thematic framework and compared with five poorly performing profiles to identify strategies for successful user engagement.

**Results:**

Profiles that were able to successfully engage large numbers of users were more active and had higher levels of interaction per user than lower-ranked profiles. Strategies used by the top ten ranked profiles included: making regular posts/tweets (median 46 posts or 124 tweets/month for top-ranked profiles versus six posts or six tweets for poorly-performing profiles); individualised interaction with users (85% of top-ranked profiles versus 0% for poorly-performing profiles); and encouraging interaction and conversation by posing questions (100% versus 40%). Uploading multimedia material (80% versus 30%) and highlighting celebrity involvement (70% versus 10%) were also key strategies.

**Conclusion:**

Successful online engagement on social networking platforms can be measured through quantitative (user numbers and interactions) and basic qualitative content analysis. We identified the amount and type of host activity as key strategies for success, and in particular, regular individualised interaction with users, encouraging conversation, uploading multimedia and relevant links, and highlighting celebrity involvement. These findings provide valuable insight for achieving a high level of online engagement through social networking platforms to support successful health promotion initiatives.

**Electronic supplementary material:**

The online version of this article (doi:10.1186/s12889-015-1396-z) contains supplementary material, which is available to authorized users.

## Background

Online social networking platforms (SNP) such as Facebook and Twitter have grown rapidly in popularity since the birth of Web 2.0 applications. Facebook grew from over 300 million active users in 2009 [1] to 1.1 billion as of March 2013 [2]. Twitter has over 200 million active users creating over 400 million tweets each day [3]. A defining feature of Web 2.0 technology is interaction between end-users and the host through user-generated content. SNP allow public and private messaging, photo, video and other content sharing, live updates, the formation of groups and the use of other applications such as games, quizzes and polls [4-6]. The two-way information flow means users can engage and be content creators, rather than simply passive recipients of information as with Web 1.0 technology [7]. This online social engagement enables individuals to build online communities through shared interests and identities. The extensive reach of sites such as Facebook and Twitter, along with their interactive functions, offers huge potential in terms of delivery of health promotion messages [7-9].

A key goal of health promotion is to enable people to increase control over and improve their health [10], often through individual behaviour change. Interactive health promotion campaigns that encourage participation and engagement, rather than providing a one-way flow of information, are reported to have greater potential to enhance behaviour change [11-13]. Engagement demonstrates awareness and contemplation, and promotes deep learning and understanding [14]. Contemplation is considered one of the initial stages in the process of behaviour change, leading to preparation and finally to action [15]. Results from systematic reviews [16-18] and a meta-analysis [19] demonstrate that internet- or computer-based interventions have the potential to not only affect knowledge, attitudes, intentions and social norms (determinants of health behaviour change) but effect health behaviour change in several areas. Although these studies did not look specifically at SNP, it seems reasonable that SNP that promote a high degree of engagement in health issues could act as important catalysts in achieving behaviour change.

Outside the field of health promotion, social marketing has embraced Web 2.0 technology. Social marketing aims to influence behaviour in a beneficial way [20], achieving audience engagement through commercial marketing techniques. Numerous case studies exploring the use of Facebook and Twitter for social marketing have described innovative strategies that successfully engage audiences [21,22]; however, many do not define how they measure online engagement or the success of campaigns. Despite a growing number of examples of successful social media campaigns in the corporate sector, empirical methods to measure engagement and identify strategies for successful engagement have lagged behind.

Our 2010 systematic review of the use of social networking sites for sexual health promotion [23] found many organisations involved in sexual health promotion *are* using SNP, but the extent of activity on these sites varied considerably and the vast majority of activities were not reported in the scientific literature [24]. Pedrana et al’s (2013) evaluation of a sexual health promotion campaign demonstrated the potential of SNP for sexual health promotion among gay men [25]. The evaluation measured reach and engagement and found that the ‘webisode’ format of video uploads was an effective way to deliver health promotion information. Pedrana et al. asserted that the combination of education and entertainment (or ‘edutainment’ [26]) was a key element of success for this intervention, along with targeted Facebook advertisements to attract users. They attributed ongoing user interest and interaction to user-perceived quality of content and the video format [25]. Online interaction can indicate peer-validation or acceptance of a topic/post, in turn influencing others’ behaviour, and providing the potential for increased reach and interaction. Other researchers have identified reach and interaction metrics as important for monitoring the success of social media interventions [8,25,27].

Several researchers have recently identified features of web information systems likely to enhance engagement and to improve their health promotion potential [28-30], including primary task support (e.g. message tailoring, personalization), dialogue support (e.g. praise, reminders, rewards), system credibility (e.g. trustworthiness, real-world feel), and social support (e.g. social learning, normative influence); however, few have been validated using SNP. Although combined literature on health promotion, social media and consumer engagement suggest that increasing reach and user interaction are crucial elements of success, no consensus exists on the key factors required to achieve this. To guide the development of health promotion activities in this emerging field, we aimed to establish a method for measuring successful online engagement, to identify Twitter and Facebook profiles that successfully attracted and engaged users, and characterise key strategies used to achieve this success.

## Methods

### Study design and search strategy

This study was a prospective descriptive analysis of Facebook and Twitter profiles drawn from our previous systematic examination of SNP for sexual health promotion carried out in 2010 [23]. The search strategy (described in detail previously) [23] used databases of published scientific literature, SNP search functions, and electronic sources of grey literature to identify SNP involved in sexual health promotion.

### Inclusion criteria

**Figure 1:**
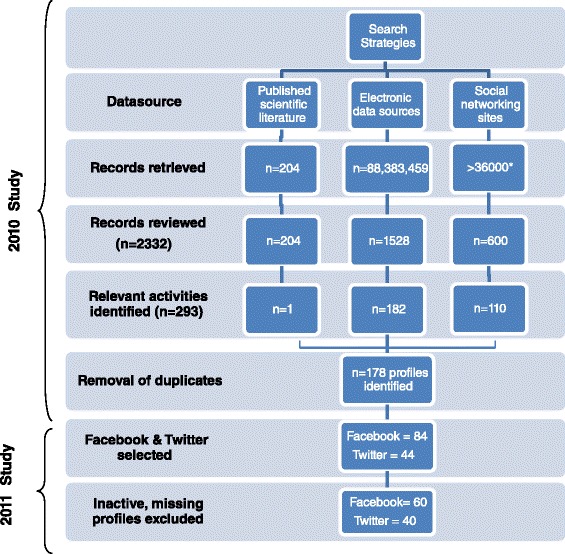
**Search results and selection of Facebook and Twitter profiles.** * Facebook does not supply total number of records found in a search, thus the total number of records retrieved from the searches in social networking sites is not available

Search results and inclusion criteria for SNP in this analysis are described in Figure 1. Of the 178 SNP identified in the previous review, we included only Facebook and Twitter profiles (n = 130) given that they are the most popular platforms globally [31] and the most enduring over the follow-up period. Profiles were excluded if they were no longer available online or were ‘inactive’; that is, if the host organisation had not posted in the 30 days prior to data collection (September 1, 2011). We performed quantitative and qualitative analyses on the selected 100 Facebook and Twitter profiles that remained active and accessible.

### Data extraction and key metrics

We examined one month of content from the 60 Facebook profiles (August 6 to September 5, 2011) and 40 Twitter profiles (September 4 to October 4, 2011). Two researchers extracted and recorded relevant quantitative metrics (see Table 1). We generated PDF images of web content covering the period of interest to enable researchers to revisit content. Data were analysed in Stata 11 (StataCorp., Texas, 2009).

**Table 1:** **Facebook and Twitter metrics**

**Categories**	**Metrics**
**Reach**	Facebook: Number of total Facebook page ‘likes’ (users)
	Twitter: Number of followers (users)
**Interaction**	Facebook:
	i. Number of comments made on posts
	ii. Number of ‘likes’ of posts and of comments
	Twitter:
	i. Number of ‘retweets’ by users (both of host tweets - ‘first degree retweets’, and of host retweets - ‘second degree retweets’)
	ii. Number of ‘replies’ by users
	iii. Number of ‘mentions’ by users

We defined ‘reach’ as the size of the user base, that is, the number of Twitter followers or Facebook ‘page likes’. (‘Liking’ an organisation’s Facebook page or following their Twitter account is equivalent to subscribing to the organisation’s status updates or tweets).

‘Interaction’ was used to indicate user engagement, and was defined as any interaction that occurred between users or between a user and the host of the profile. To create a measure of overall interaction, we summed all interaction metrics (Table 1) for each profile to get a ‘total interaction score’. We also calculated mean interactions *per user* for each profile by dividing the interaction score by the number of users.

### Identifying Facebook and Twitter profiles with high levels of engagement

Our measure of ‘engagement’ combined the size of the user base (reach) and the level of interaction of the users. We considered profiles with the largest user bases and highest levels of interaction to have the highest levels of online engagement. To measure engagement, we ranked all profiles within each category of reach and interaction, with profiles that had the greatest reach and highest interaction scores being ranked highest. We then summed the reach and interaction ranks for each profile and ranked these sums again to calculate an overall engagement success ranking.

### Identifying factors and strategies associated with engagement

We used quantitative and qualitative methods to compare top-ranked and low-ranked profiles to identify factors and strategies associated with engagement.

#### Quantitative data analysis

We compared the levels of host activity – defined as the number of Facebook posts or number of tweets or retweets by the host organisation – for top ten ranked profiles with all other profiles using Kruskal-Wallis tests for assessing differences between medians. This was also done for the levels of interaction per user. We explored associations between reach, activity and interaction to understand how they related to success and each other. Spearman rank correlations were used to assess the following relationships within the top ten successful profiles and within all other profiles:

- reach and interaction per user;
- activity and interaction;
- activity and interaction per user; and
- activity and reach.

#### Qualitative data analysis

The qualitative analysis involved two parts: 1) an examination and classification of the target audiences, primary activities and purposes of the top ten profiles and; 2) an examination of the specific strategies used by the top ten and bottom five organisations to determine the activities that distinguished levels of engagement. For the second part, we developed a simplified strategy for web content analysis (informed by the work of others [32,33]), given that the large volume of content made traditional in-depth content analysis unfeasible. Two stages of content analysis were involved: development of a framework for analysis, and application of the framework to the Facebook and Twitter content.

1. *Development of the content analysis framework*
  Broad categories or thematic groupings of the strategies (interactive features and activities) used by the various profiles were developed iteratively. We briefly examined top ten profiles, and constructed a preliminary framework of strategies based on what was seen in these profiles. Through consultation with the research team, further modifications were made to the framework. To test its utility and reliability, two researchers applied the refined framework to a sample of three Facebook and three Twitter profiles. The researchers identified strategies used by the profiles and categorised them according to the framework. We then compared results, measuring inter-rater reliability at 70%. After further consultation and consensus, and final modification, the framework consisted of 16 Facebook and 17 Twitter strategies grouped into seven categories. Two researchers analysed a further 200 tweets and 30 days of Facebook pages to further validate the framework. They found discrepancies in only two strategies (easily resolved with discussion), giving close to 90% inter-rater reliability.
2. *Application of the content analysis framework*
  One researcher completed the content analysis of the top ten and bottom five Facebook and Twitter profiles. She evaluated each profile against the framework and counted the strategies observed. Any content that was difficult to categorise was discussed with two other researchers and consensus reached. She calculated the percentage of top ten and bottom five profiles demonstrating each strategy.

### Ethics

Ethics approval was received from The Alfred Health Human Ethics Committee (Project 6/15).

## Results

From the original sample of SNP involved in sexual health promotion [23], we identified 84 Facebook and 44 Twitter profiles. Sixty Facebook profiles and 40 Twitter profiles were included in the analyses; of the other 28, nine were no longer available online and 19 were inactive (Figure 1).

### Identifying Facebook and Twitter profiles with high levels of engagement

Eight host organisations had both Facebook and Twitter profiles in the top ten ranking for engagement, demonstrating that some organisations were able to achieve success across both platforms.

Table 2 illustrates reach and interaction metrics for top ten ranked profiles and all other profiles. For the top ten Facebook profiles, the median number of users was 15,156 compared with 560 for all other profiles; and for Twitter, 8,558 compared with 852. The median total number of user interactions for top ten Facebook profiles was 1325 versus 16 for all other Facebook profiles, and for Twitter, 937 versus 37. The most common types of interaction in the top ten profiles were Facebook likes of posts, and Twitter follower retweets, however across all Twitter profiles, ‘mentions’ were the most common type of interaction.

**Table 2:** **Reach and interaction in top ten ranked and less successful profiles**

		**Top ten profiles**	**All other profiles**
		**Median (IQR)**	**Median (IQR)**
**Facebook metrics**	**Reach:**	15156 (10598, 115782)	560 (293, 1147)
	Number of users		
	**Interaction:**		
	No. Likes	1155 (229, 3273)	12 (3, 36)
	No. Comments	125 (47, 377)	2 (0, 6)
	No. Likes of comments	47 (19, 93)	0 (0, 2)
	Total interaction score	1325 (379, 3702)	16 (4, 41)
**Twitter metrics**	**Reach:**		
	Number of users	8558 (4886, 35073)	852 (317, 1372)
	**Interaction:**		
	No. first degree user retweets	368 (159, 415)	8 (2, 26)
	No. second degree user retweets	93 (24, 280)	0 (0, 31)
	No. of user replies	52 (38, 65)	6 (1, 14)
	No. of mentions by user	270 (180, 383)	21 (4, 39)
	Total interaction score	937 (543, 1209)	37 (7, 158)

### Identifying factors and strategies associated with engagement - quantitative analysis

Interaction per user and activity metrics are shown in Table 3 for top ten ranked profiles and all other profiles. The level of interaction per user was significantly greater for top ten ranked Facebook profiles than all other profiles (p = 0.05), but no significant difference (p = 0.57) existed between top ten Twitter profiles and all other profiles. Top-ranked profiles were more active than other profiles: they had a median of 46 posts per month compared with nine for Facebook profiles (p < 0.01), and 124 tweets per month versus 29 for Twitter profiles (p < 0.01).

**Table 3:** **Comparing interaction per user and activity in top ten ranked and less successful profiles**

		**Top ten profiles**	**All other profiles**	p-value*
		**Median (IQR)**	**Median (IQR)**	
**Facebook**	**Interaction per user:**	0.07 (0.04, 0.1)	0.03 (0.01, 0.06)	0.05
	**Activity:**			
	No. Posts	46 (24, 72)	9 (5, 19)	<0.01
**Twitter**	**Interaction per user:**	0.09 (0.03, 0.11)	0.04 (0.01, 0.13)	0.57
	**Activity:**			
	No. Tweets	124 (76, 220)	29 (10, 49)	<0.01

*Kruskal-Wallis Test.

#### Exploring associations between reach, interaction and activity

Results for this section are shown in Table 4. For the top ten ranked Facebook profiles, the association between host activity (posts) and interaction was not statistically significant (Spearman’s rho 0.34, p = 0.34). For the top ten ranked Twitter profiles, we found a no significant association between the host activity (tweets) and interaction (Spearman’s rho 0.14, p = 0.70). However, for non-top ten ranked Facebook and Twitter profiles, host organisation activity was strongly associated with interaction (Spearman’s rho 0.59, p < 0.001; and 0.64, p < 0.001 respectively).

**Table 4:** **Exploring associations between reach, interaction and activity in top ten ranked and less successful profiles**

	**Correlations between:**	**Spearman’s Rho**	**Spearman’s Rho**
		**(p-value) for top 10 ranked profiles**	**(p-value) all other profiles**
**Facebook**	Activity & interaction	0.34 (0.34)	0.59 (<0.001)
	Activity & interaction per user	0.59 (0.07)	0.53 (<0.001)
	Activity & reach	−0.07 (0.85)	0.20 (0.16)
	Reach & interaction per user	−0.11 (0.75)	−0.17 (0.23)
**Twitter**	Activity & interaction	0.14 (0.70)	0.64 (<0.001)
	Activity & interaction per user	0.85 (<0.001)	0.66 (<0.001)
	Activity & reach	−0.5 (0.13)	0.23 (0.21)
	Reach & interaction per user	−0.76 (0.01)	−0.04 (0.82)

Host activity and interaction per user were strongly positively correlated for both top ten ranked Facebook and Twitter profiles (Spearman’s rho 0.59, p = 0.07; and 0.85, p < 0.001, respectively), as well as for all other profiles (Spearman’s rho 0.53, p < 0.001; and 0.66, p < 0.001). The correlation was strongest within the top ten Twitter profiles.

Host activity and reach in the top ten ranked Facebook profiles were not associated (Spearman’s rho −0.07, p = 0.85). For top ten ranked Twitter profiles, a negative association was not statistically significant (Spearman’s rho −0.5, p = 0.13). For non-top ten Facebook and Twitter profiles, only a weak and non-significant association existed between activity and reach (Spearman’s rho 0.20, p = 0.16; and 0.23, p = 0.21 respectively) (Table 4).

The correlation between reach and interaction per user was very weak for top ten ranked Facebook profiles (Spearman’s rho −0.11, p = 0.75) and all other profiles (−0.17, p = 0.23) (Table 4). Similarly, for all other Twitter profiles, there was no correlation between reach and interactions per user (Spearman’s rho −0.04, p = 0.82) (Table 5). For top ten ranked Twitter profiles however, there was a strong negative correlation between reach and interaction per user (Spearman’s rho −0.76, p = 0.01).

**Table 5:** **Characteristics and strategies employed by successful and unsuccessful profiles**

**Broad strategy**	**Specific strategy**	**Facebook**		**Twitter**	
		**Top 10 (%)**	**Bottom 5 (%)**	**Top 10 (%)**	**Bottom 5 (%)**
**Direct engagement with users**	Acknowledges/supports followers/friends	60	20	90	0
	Host replies directly to user	100	0	70	0
**Links in with established user bases/cross promotion with other organisations**	Hash Tags	n/a	n/a	100	20
	Retweets content from other organisations/individuals	n/a	n/a	100	40
	Links to campaigns/events	80	20	100	60
	Host website has link to Twitter/Facebook profile	80	80	80	40
**Encourages interaction/fosters online community amongst users**	Polls/quizzes/surveys	50	20	50	0
	Poses questions and/or initiates conversation	100	60	100	20
	Allows users to post	60	0	n/a	n/a
**Makes content broadly relevant and engaging**	Links to relevant content/organisations	100	100	100	80
	Uses humour	20	100	20	0
	Posts time-relevant content	90	60	100	80
	Involves expert/trusted source	30	0	80	20
	Makes use of multimedia uploads (video/photos/audio)	70	40	90	20
	Highlights celebrity/ high profile involvement in the issue/cause	60	20	80	0
**Increases following/visibility**	Incentives/prizes/competitions	20	40	50	60
	Encourages posting, sharing and tagging of photos	40	0	20	0
**Regular activity**	Regular tweets/posts (median no. per month (IQR))	46 (24, 72)	6 (3, 7)	124 (76, 220)	6 (3, 6)

### Identifying factors and strategies associated with engagement – qualitative analysis

#### Description of top ten profiles

Most of the top ten Facebook and Twitter profiles (70%) had a sexual and reproductive health and/or HIV/AIDS focus. This was expected given that our original search included profiles ‘involved in sexual health promotion’. Fewer profiles (30%) had a general health focus. All top ten profiles were part of ongoing social networking profiles rather than short-term campaigns. The target audience varied: 40% of top ten ranked profiles had a general target audience, 35% targeted people living with or affected by HIV/AIDS, 20% targeted women, and only one profile specifically targeted young people. The majority of profiles (80%) used sites for the purpose of sharing or disseminating health information – e.g. about health conditions, screening or testing, wellness strategies, research and statistics, health products, or answering specific health-related questions. About half of Facebook and Twitter profiles (40% and 50% respectively) utilised their profile for advocacy or campaigning, such as lobbying activities, keeping users informed on current policy, relevant news and political events related to specific health or health care issues. Half also used their profiles to foster online communities to increase peer or social support, e.g. enabling individuals with similar interests/concerns to connect and communicate (see Additional file 1).

#### Strategies used by profiles with high levels of engagement

Table 5 shows the results of the qualitative content analysis: the various strategies used by the top ten ranked versus bottom five ranked Facebook and Twitter profiles.

Strategies more common among profiles with larger and interactive user bases included: regular host activity; direct, individualised interaction with users; and posing questions to encourage interaction and conversation. Top-ranked profiles were more likely to upload multimedia and highlight celebrity or high profile involvement in the cause to make content broadly relevant and engaging. (Some examples of these strategies are presented in Additional file 2).

## Discussion

We developed a framework to systematically identify and assess Facebook and Twitter profiles that could successfully engage audiences from a large database of profiles broadly involved in sexual health promotion. We based our definition of engagement on two commonly-used metrics, reach and interaction, and defined successful engagement as Facebook and Twitter profiles with large reach and high numbers of total interactions with and between users. Profiles with a high level of engagement (top ranked) were more active than profiles with poorer engagement and demonstrated greater levels of interaction per user. In an exploratory analysis, we compared top-ranked and low-ranked profiles to assess associations between measures of reach, interaction and host activity. For top-ranked profiles, the association between host activity and interaction per user was greater than for low-ranked profiles. Qualitative analysis identified key strategies common to top-ranked profiles that achieved a high level of engagement, including regular and individualised interaction with users, encouraging conversation, uploading multimedia and relevant links, and highlighting celebrity involvement. These findings highlight the importance of tailoring activity to encourage interaction and ensure ongoing reach and engagement of target audiences. Organisations using SNP can utilise our results, whether it be for disseminating health information, campaigning and advocacy around a certain health issue, or for fostering online communities to provide social support.

### Characteristics of success

Previous work in this area has identified common key metrics, including reach and interaction, which can be used to help measure, monitor and inform the success of such social media interventions [8,25,27]. However, relatively few have actually gone beyond using these metrics to measure success, to identify features or strategies that could enhance the health promotion potential of web information systems and online applications [28-30]. We chose reach and interaction as our key metrics to measure online engagement by SNP-based sexual health promotion campaigns. Through quantitative methods, we found that higher levels of host activity (posts and tweets) and greater levels of interaction per user characterised profiles with high levels of engagement. We then explored associations between SNP metrics to gain more insight into achieving increased reach and interaction. We found a strong positive correlation between host activity and levels of interaction per user, especially for top ten Twitter profiles. This suggests that it is important to maintain host-initiated activity on SNP to encourage meaningful interactions both between the host and users, but also between users, in order to ensure ongoing engagement of the target audience. However, it is unlikely that high levels of activity alone are enough to ensure interaction, and consideration should be given to the *type* of activity that is most likely to promote interaction with and between users, and what type of activity generates certain kinds of interaction. For example, organisations may want to go beyond simply reaching people with messages, and focus more on drawing users into conversations and debate on a topic, that is, considering the *content value* of posts and tweets [34].

For top-ranked Facebook profiles, there was no association between the reach and the level of interaction per user. That is, the likelihood of a Facebook user interacting is not related to the number of users/friends of the profile. Interaction per user may be more related to host activity (as above) or the target audience or purpose of the profile, e.g. forming an interactive online community versus broad dissemination of health information. For top-ranked Twitter profiles, there was a strong negative correlation between reach and interaction per user: as the number of users increased, the level of interaction per user decreased. This may be reflective of the types of users and how and why people use Twitter. It suggests that the top-ranked Twitter profiles are broadcasting to large numbers of users, with many users being passive receivers of the information rather than interacting and engaging in conversation. Twitter is commonly used to post messages to many users, to follow live updates during major events (sporting, cultural, news), or to receive regular brief updates from large organisations or celebrities. Conversation on Twitter is generally limited in comparison to Facebook, which is designed to support social networks of friends or individuals with common interests, allowing them to share content by posting photos, links or creating stories and conversations. Organisations should reflect on their health promotion goals and target audience when selecting an appropriate SNP platform; for some reaching a large audience with brief messages is more important, but others seek primarily to engage a particular group or community and generate discussion.

### Key strategies to reach and engage users

Through the simplified qualitative content analysis of top-ranked and low-ranked profiles, we identified key strategies that characterised successful online engagement: making regular posts; directly engaging with users through individual responses and acknowledgment; encouraging interaction and conversation by posing questions; utilising multimedia uploads and relevant links; and highlighting celebrity involvement. Other researchers have identified similar strategies [27-30]. Preece and Shneiderman (2009)analysed online social activities and developed a framework of ‘usability’ and ‘sociability’ factors that they believed motivated online social participation [27]. They described how organisations could harness usability factors such as frequent updates and posting interesting, relevant and well-presented content to ensure users could easily engage with their content. Similarly, they identified sociability factors such as using charismatic leaders or respected authorities, and enabling development of user ‘visibility’ (an online social presence), which increased the chances of user engagement [27]. Others have found that acknowledging users’ contributions enables them to stand out or gain ‘digital status’ and is a key motivator for online contribution or interaction [35,36]. Utilising these key strategies effectively requires dedicated resources and careful planning, design and monitoring [24,37]. In our research, top ten profiles appeared to be part of long-term ventures – organisations’ overall communication strategies – rather than short-term campaigns or initiatives, highlighting the importance of maintaining an online presence and working towards building online communities engaged with a cause/issue. It is clear from both our review and the work of others that in order to deliver successful interventions through SNP, hosts use various key strategies to take advantage of the different functionalities of the platforms, as well as improving the quality and utility of content being delivered to their target audience.

### Considerations for future projects utilising SNP for health promotion

#### Defining successful online engagement

When defining and measuring online engagement we weighted reach and interaction metrics equally, however it is likely that the relative merits of each vary according to organisations’ objectives. For some, simple exposure to information through SNP could be enough to reap meaningful benefit, whereas for others it may be more important to have interaction, either between users or with the host organisation in order to promote program participation and ultimately gain benefit [13]. It may be more beneficial for organisations to examine the different metrics separately to enable them to better target different strategies based on their goals (to increase reach or interaction, or both). When measuring engagement, we combined all interaction metrics into one interaction score; although in reality different forms of interaction may indicate different levels of engagement. For example, a simple ‘like’ or ‘retweet’ (the most common forms of interaction in our study) requires only a simple click of a button, and essentially acts to share content with the user’s networks. However, a ‘comment’, ‘reply’ or ‘mention’ requires the user to formulate a response, indicating deeper thought and engagement. Thus our definition of engagement was limited in its ability to identify profiles better at promoting interaction on a superficial versus a more in-depth level. Neiger and colleagues (2013) have developed an evaluation hierarchy for social media engagement, representing different levels of engagement that could be applied when defining successful engagement [13]. Another way to describe online engagement through social media is ‘influence’, with reach and different interactions representing different potential levels of influence of a host on an audience [34].

#### Quality of posts and tweets and types of users

One aspect of interaction we were unable to measure was the quality or the content of the post/tweet; for example, did successful profiles post better quality content or more humorous content than less successful profiles? This should be a consideration for both future researchers and future intervention planners who will need to consider not only the content itself but the quality and style in which it is delivered. This has worked successfully for sexual health promotion through the ‘Being Brendo/Queer as Fxxk’ project, in which a Facebook page targeting men who have sex with men (MSM), through the delivery of short, humorous and educational ‘webisodes’ that the target audience found to be personally relevant, was able to attract a large and active user base [24,25]. A second ‘Facespace’ page that targeted the young heterosexual population with similar messages was less successful. Evaluation of both projects attributed this to the different approach with regards to both the type and delivery of the content, whereby the MSM arm, designed as a drama series (and more refined and expensive), was more successful at engaging and entertaining its target audience. Organisations wanting to replicate this success need to identify common interests and shared identities within their target audiences that can be utilised to connect different types of users and promote interaction. Recent work looking at the different ‘types’ of social media users found that the majority of users tend to be passive, using social media to seek information or keep up with the online activities of others, without sharing content, responding or ‘posting updates’ themselves [20,38]. Understanding the various types of users and their motivations for engaging will help organisations to identify and understand different user characteristics, motivations for engaging and types of engagement that are likely to occur on social media, which will enable them to better engage and influence their target audiences.

## Limitations

When we conducted the review on which this work is based, there was (and remains) a lack of best practice guidelines for systematic searches of social networking sites [23,39]. Large amounts of Facebook and Twitter content precluded the examination of strategies using traditional qualitative thematic analysis. The framework of strategies that we developed enabled a modified content analysis; however, future researchers could examine the actual content of user interactions to explore what kind of host activity elicits the deepest or most valuable user interaction, or the widest dissemination (e.g. posts or tweets that ‘go viral’). Although there are more sophisticated and complex methods for analysis of social media content, such as social media mining and commercially available tools that can code data [39,40], health promotion practitioners and others can easily apply our methods to both Facebook and Twitter platforms. Furthermore, some suggest that manual coding of content is ideal given the unconventional text typically used on social media [39]. Although our sample of SNP was taken from an original search that focused on sites involved in sexual health, the selected metrics and methods described can be applied to social media in general.

The rapidly evolving functionality and features of Facebook and Twitter mean that other, more novel strategies may currently be in use to increase the reach and interaction on these platforms. For example, the Facebook ‘Share’ feature was not utilised by all profiles we examined, and its functionality has recently altered to enable content to be visible in timelines and newsfeeds of users and their networks [41]. Other recent developments include the ability to post images as comments on Facebook and the use of hashtags on posts. Twitter has made alterations to the ‘tweet’ and ‘follow’ buttons to further direct and drive traffic, and has enabled ‘embedded’ tweets on websites to drive traffic to the SNP. Such new features complement the key strategies we have identified, which focus on the type of host activity associated with success more than the functionality of the platforms. Therefore, although data collection occurred in 2011, our key strategies should remain very relevant into the future, with the focus being on how new functions can support these key strategies. Although all profiles we examined were involved in sexual health, it is likely that the key strategies identified can be applied universally to increase online engagement. Nevertheless, given that our top ten profiles were all from large, well-resourced organisations, mostly with existing public profiles, achieving success with smaller, newer profiles might require a modified approach.

## Conclusion

We offer one way to measure successful engagement on social networking platforms through quantitative (user numbers and interactions) and basic qualitative content analysis. Key strategies associated with a high level of engagement included regular individualised interaction with users, encouraging conversation, uploading multimedia and relevant links, and highlighting celebrity involvement. Consideration should be given to whether achieving greater reach or greater interaction (or both) is important, and which platforms are most suited to achieving these goals. Future research should determine which strategies are most effective for reaching, engaging and retaining certain types of users; what makes a post/ tweet ‘go viral’; the kind of content that promotes the greatest interaction; and what other aspects or functionalities of SNP can enhance engagement. These findings provide valuable insight for supporting successful online health promotion campaigns. We have laid the foundation for further research; as the field matures and more evidence emerges, our results will represent an adaptive platform from which online engagement can be evaluated.

## Additional files

Additional file 1:
**‘Primary activities of top ten Facebook and Twitter profiles’.** This table categorises the main activities of SNP that we investigated, and provides a description of each. Additional file 2:
**‘Examples of key strategies used on SNP’.** This table gives real examples of organisations using the key strategies we identified.

## Abbreviation

SNP: Social networking platforms
