# Supplementary material for: The use of social networking platforms for sexual health promotion: identifying key strategies for successful user engagement
Source: BMC Public Health. 2015 Feb 6;15:85. doi: 10.1186/s12889-015-1396-z (PMC4340797; doi:10.1186/s12889-015-1396-z)
Supplement: Additional file 1: — ‘Primary activities of top ten Facebook and Twitter profiles’. This table categorises the main activities of SNP that we investigated, and provides a description of each. [file 12889_2015_1396_MOESM1_ESM.doc]

# Additional file 1. Primary activities of top ten Facebook and Twitter profiles

| **Primary activities of SNP profiles** | **Description** |
| --- | --- |
| 1. Sharing of health information/ health promotion | Provision of health-related information e.g. about a health condition, screening or testing information, wellness strategies, prevention strategies, research and statistics, health products, answering specific health-related questions etc. |
| 1. Advocacy/ campaigning | Lobbying activities, keeping users informed on current policy, relevant news and political events related to specific health or health care issues etc. |
| 1. Offering peer/ social support or an online community | Enabling individuals with similar interests/ concerns to connect and communicate. |
