# Supplementary material for: The use of social networking platforms for sexual health promotion: identifying key strategies for successful user engagement
Source: BMC Public Health. 2015 Feb 6;15:85. doi: 10.1186/s12889-015-1396-z (PMC4340797; doi:10.1186/s12889-015-1396-z)
Supplement: Additional file 2: — ‘Examples of key strategies used on SNP’. This table gives real examples of organisations using the key strategies we identified. [file 12889_2015_1396_MOESM2_ESM.doc]

# Additional file 2. Examples of key strategies used on SNP

| **Broad Strategy** | **Specific strategy** | **Example** |
| --- | --- | --- |
| Direct engagement with users | Acknowledges/ supports followers/ friends | *‘Thanks @jljacobson for helping to spread the word and the great article on…’* |
|  | Host replies directly to user | *‘Sorry to hear about your ills. The most likely cause for your symptoms is a virus. viral infections…’* |
| Encourages interaction / fosters online community | Poses questions and encourages conversation | *‘New web-based intervention program teaches teen bystanders to ‘get in the way’. What do you think?’* |
| Makes content broadly relevant and engaging | Makes use of multimedia (video/ photos/ audio) | *‘beautiful video…thanks for sharing! RT@calvinfinch Great music video!...* [watch video] |
|  | Involves expert/ trusted source | *‘Dr Archelle Georgiou talks about ovarian cancer: http://...’* |
|  | Highlights celebrity/ high profile involvement in the issue/ cause | *‘Lil B Calls on Fans To Go Get Tested In New Song ‘I Got AIDS’ on mtv.com’*  *Zimbabwe President Robert Mugabe Urges Men to Take Larger Role in HIV Prevention’* |
